# RuO_2_ pH Sensor with Super-Glue-Inspired Reference Electrode

**DOI:** 10.3390/s17092036

**Published:** 2017-09-06

**Authors:** Wade Lonsdale, Magdalena Wajrak, Kamal Alameh

**Affiliations:** 1 Electron Science Research Institute, Edith Cowan University, Joondalup, WA 6027, Australia; k.alameh@ecu.edu.au; 2 School of Science, Edith Cowan University, Joondalup, WA 6027, Australia; m.wajrak@ecu.edu.au

**Keywords:** ruthenium oxide, solid-state pH sensor, polyvinyl butyral, silicon dioxide, differential-type pH sensor

## Abstract

A pH-sensitive RuO_2_ electrode coated in a commercial cyanoacrylate adhesive typically exhibits very low pH sensitivity, and could be paired with a RuO_2_ working electrode as a differential type pH sensor. However, such sensors display poor performance in real sample matrices. A pH sensor employing a RuO_2_ pH-sensitive working electrode and a SiO_2_-PVB junction-modified RuO_2_ reference electrode is developed as an alternative high-performance solution. This sensor exhibits a performance similar to that of a commercial glass pH sensor in some common sample matrices, particularly, an excellent pH sensitivity of 55.7 mV/pH, a hysteresis as low as 2.7 mV, and a drift below 2.2 mV/h. The developed sensor structure opens the way towards the development of a simple, cost effective, and robust pH sensor for pH analysis in various sample matrices.

## 1. Background

The use of RuO_2_ films for the manufacture of solid state potentiometric pH sensors has several advantages, namely Nernstian pH sensitivity, insolubility over a wide pH range, good reproducibility, low hysteresis, and reduced cost (in comparison to the more commonly studied IrO_2_ films) [1,2,3,4]. The pH sensing properties of RuO_2_ films have been reported by numerous groups [5,6,7]. Briefly, RuO_2_ undergoes the following redox reaction:

(1) $${\mathrm{RuO}}_{x}{\left(\mathrm{OH}\right)}_{y}+{\mathrm{ze}}^{-}+{\mathrm{zH}}^{+}↔\;{\mathrm{RuO}}_{x-y}{\left(\mathrm{OH}\right)}_{y+z}$$

where the electrode’s potential, *E*, in mV and at 22 °C, is given by the Nernst equation, which simplifies to:

(2) $$E=E^{0}-58.6\mathrm{pH}$$

Radio frequency magnetron sputtering (RFMS) is a convenient technique for the deposition of thin films with well-controlled thickness and stoichiometry [8]. This technique is therefore attractive for the development of RuO_2_ pH sensors featuring high purity and reproducible performance.

Metal oxide pH sensors are commonly paired with quasi Ag|AgCl reference electrodes for potentiometry, since they are simple to construct [9,10,11]. However, quasi reference electrodes are not always suitable for application in samples due to their cross sensitivities. For example, an Ag|AgCl electrode is sensitive to the concentration of Cl^−^ ions in solution, according to the following equations:

(3) $${\mathrm{AgCl}}_{\left(s\right)}+e^{-}↔{\mathrm{Ag}}_{\left(s\right)}+{\mathrm{Cl}}_{\left(\mathrm{aq}\right)}^{-}$$

(4) $$E=E^{0}+\left(\frac{RT}{nF}\right)\ln\left[\alpha_{{\mathrm{Cl}}^{-}}\right]+\left(\frac{RT}{nF}\right)\ln\frac{\left[\mathrm{AgCl}\right]}{\left[\mathrm{Ag}\right]}$$

(5) $$E=E^{0}+58.6p\left[\mathrm{Cl}\right]$$

where *R*, *T*, *n*, and *F* are the universal gas constant, temperature, number of electrons, and Faraday constant, respectively. This makes such sensors difficult to use in sample solutions, where Cl^−^ concentration changes. Much research has been undertaken to develop Cl^−^-insensitive solid-state reference electrodes [12,13,14]. This is commonly achieved by adding a KCl electrolyte layer to the Ag|AgCl electrode, which results in a high concentration of Cl^−^ at the electrode’s surface and thus a stable electrode potential. KCl is used to minimize the formation of a liquid junction potential, due to the nearly equal ionic mobilities of K^+^ and Cl^−^ [15]. 

Previously, authors have reported numerous electrolyte layers and modification procedures including gels [16], fused ceramics/glasses [17,18], and other polymers [19,20]. Typically, gels suffer from short life spans and are not commonly used due to their low melting points, whilst fused glass requires high temperatures during their manufacture process. This makes polymers more attractive, since they can be drop-cast and dried at room temperature. However, polymers are more chemically reactive than glass and can be prone to interference from solvents and other agents. Electrodes based on KCl electrolyte layers also have varying lifespans depending on the rate at which KCl leaches from the electrode. Lifespans ranging from several days [15] to several months [21] have been reported. 

Other approaches for the development of solid-state pH sensor reference electrodes include the use of bronzes or similar materials that have low pH sensitivity [22,23]. However, these kinds of sensors typically exhibit poor performance due to high hysteresis, drifts, and instability caused by the reference electrode. Another approach involves modifying the pH-sensitive working electrode, so that the pH, and therefore the potential at the electrode’s surface, is constant [24]. J. Noh et al. [25] reported one such differential pH sensor based on a complex series of polymer layers over a Pt electrode. In this paper, a differential-type pH sensor, employing RuO_2_ as a pH-sensitive working electrode and RuO_2_ modified with a simple polymer layer loaded with silica as a reference electrode, is proposed and its performance is investigated experimentally. 

## 2. Preliminary Work

**Figure 1:**
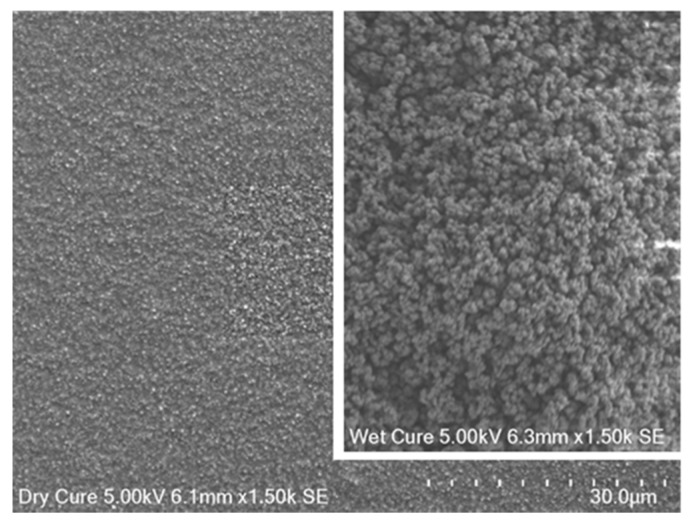
Surfaces of air-cured and pH 7-cured super-glue (inset), at the same magnification.

In this study, a RuO_2_ electrode was covered with a commercial adhesive (Loctite Super Glue—Gel Control) and, surprisingly, it showed very low pH sensitivity, so it was investigated for use as a reference electrode. However, the manufacture of this electrode was difficult to replicate. The initial electrode was manufactured on a Zensor screen-printed carbon electrode on a polyethylene terephthalate (PET) substrate, with a 500-nm thick RuO_2_ film. A 1–2 mm layer of the glue was applied over the RuO_2_ working area, and the glue was cured by placing it in a pH 7 buffer solution for 24 h. Curing in the pH 7 buffer solution was found to be essential, as a voltage reading could not be obtained for electrodes that were cured in air, indicating that a complete water tight encapsulation layer had formed over the RuO_2_ film. When cured in pH 7 buffer, an opaque white material was formed (when the electrode was dry), which quickly became clear when submerged in a liquid. This indicated that the glue cured at pH 7 possessed a porous structure. Presumably, the formation of this porous material can be attributed to the curing process, since cyanoacrylate (superglue) polymerizes when exposed to H_2_O. This likely results in rapid polymerization (and a porous structure) when the glue is cured in a solution, whereas in air the glue is able to cure slowly, forming a smooth clear-plastic layer. Figure 1 shows SEM images of the air-cured and pH 7-cured super-glue surfaces. It is obvious from Figure 1 that the air-cured glue is flatter and more uniform compared to the pH 7-cured glue, which is rough and appears to have many pores, when viewed at the same magnification. 

Replication of this electrode using DropSens ordered meso-porous carbon (OMC) substrates with 500 nm of RuO_2_ resulted in electrodes with inconsistent performance. It was noted that the Zensor-based electrode was more opaque when dried and became clear faster when hydrated, compared to the OMC electrodes. Closer examination of the Zensor electrodes when applying the glue revealed that there was an unknown chemical reaction occurring between the electrical isolation layer and the super glue, which seemed to result in a more porous structure. Zensor substrates were electrically isolated using a material that dissolved in acetone, whilst the DropSens electrodes used a solvent-resistant resin. 

When paired with a RuO_2_ working electrode, the original Zensor electrode performed well in pH buffer solutions (results shown in the following sections). However, when applied to real sample matrices, the sensor gave inaccurate results due to large shifts in potential and instability. The reason for this was not investigated; however, it could be due to the formation of an undefined liquid junction potential caused by unknown compounds in the proprietary products used in its construction. Therefore, research was undertaken to replicate this type of differential reference electrode, but using known components.

A possible explanation for the pH insensitivity caused by the superglue layer could be that the cyanoacrylate acts as a porous structure, allowing a small volume of liquid to penetrate to the RuO_2_ surface, and the fumed silica added to thicken the glue into a gel acts as a reservoir of H^+^/OH^−^ ions, due to their adsorption on the SiO_2_ surface [26]. This reservoir is able to buffer the small volume of liquid that fills the porous cyanoacrylate, resulting in a relatively stable pH and thus potential at the RuO_2_ surface. 

T. Guinovart et al. [27,28,29] reported a reference electrode that consists of a Polyvinyl Butyral (PVB) layer loaded with NaCl on an Ag|AgCl electrode. When conditioned in 3 M KCl, a nano-porous structure develops, which controls the flow of NaCl from the electrode. This results in a stable electrode potential due a controlled Cl^−^ concentration at the Ag|AgCl surface, with low liquid junction potential. Their electrodes exhibited good stability and lifetime, but were prone to some pH sensitivity below pH 4. Here, PVB was used to create a porous junction loaded with finely ground SiO_2_. When placed over a RuO_2_ electrode, this junction resulted in relatively stable potential between pH 1.5 and 12. When paired with a RuO_2_ working electrode, this differential-type pH sensor exhibited a performance comparable to a commercial glass pH sensor in some common sample matrices. 

## 3. Method

### 3.1. Working Electrode Fabrication

Several pH-sensitive working electrodes were manufactured, as previously reported in Reference [30], with the exception that in this work RuO_2_ thickness was only 500 nm. Amorphous thin-films of RuO_2_ were obtained by RFMS deposition at room temperature onto 4-mm diameter OMC contacts of Dropsens (DRP-110OMC) electrodes, isolated via shadow masking. RuO_2_ was deposited from a RuO_2_ target (99.95% purity) using 100 W sputter power with an 80:20 Ar:O_2_ process gas ratio at 1 mTorr chamber pressure. 

### 3.2. Reference Electrode Fabrication

The pH-insensitive reference electrodes were manufactured by sputtering either 500 nm of RuO_2_ or 500 nm of Ag using the procedure as above; however, Ag was sputtered from an Ag target (99.99% purity) using 70 W sputter power in an Ar plasma at 1 mTorr chamber pressure. A well was created around each electrode working area by gluing (Loctite Super Glue—Gel Control) an acrylic ring (5 mm internal diameter, 7 mm outside diameter, 5 mm height) that was made using a laser cutter/engraver. Once the glue had completely dried, silver electrodes were chlorinated with 50 mM FeCl_3_, until a uniform brown AgCl layer had formed; meanwhile, RuO_2_ electrodes were hydrated in pH 7 buffer for 48 h. The silver electrode-wells were filled with 50 mg of KCl and then topped with a total of 250 μL of PVB^NaCl^ solution over three aliquots, electrodes were allowed to dry overnight between each addition. The RuO_2_ electrode-wells were filled with either 50 mg of ground SiO_2_ and topped with a total of 250 μL of PVB^Sio2^ solution, or with 25 mg of ground SiO_2_, 25 mg of KCl and topped with 250 μL of PVB^Sio2+KCl^ solution. PVB solutions were prepared by mixing the reagents shown in Table 1, together in a sealed vial, after which they were homogenized using an ultrasonic bath until uniform (approximately 30 min). This resulted in three different electrode types, namely, AgCl-KCl, RuO_2_-SiO_2_, and RuO_2_-SiO_2_-KCl, along with one glued-RuO_2_ reference electrode, which consisted of 500 nm RuO_2_ on a Zensor screen-printed carbon electrode coated in Loctite Super Glue—Gel Control and cured in pH 7 buffer for 24 h, as well as a quasi Ag|AgCl electrode.

### 3.3. Potentiometric Measurements 

A Keysight Technologies (Santa Rosa, CA, USA) 34410A digital multimeter was used to record the potential between the working and reference electrodes [8]. The potential was recorded for 180 s at 1 s intervals using number of power line cycles (NPLC) set to 1, operating in the High-Impedance mode. For calculations, the last 30 data points were averaged from each potential recording to produce individual measurements (this avoided the rapid shift that typically occurs during the first 30 s of recording due to electrode equilibration). These measurements were then used to calculate the sensitivity, *E*^0^, hysteresis, and drift of the sensors [10]. The hysteresis was calculated using the difference between consecutive measurements at pH 12 [30], while electrode drift was calculated using the slope of the line-of-best-fit for the data at pH 12 over the measurement period [30]. Electrode reaction time was defined as the time taken to reach within 3 mV (i.e., 0.05 pH) of the stable potential. All measurements were made in triplicate at 22 °C in commercial pH buffers (Rowe Scientific, Sydney, Australia) and error bars represent the 95% confidence intervals. 

## 4. Results and Discussion

### 4.1. Reference Electrode Performance

**Figure 2:**
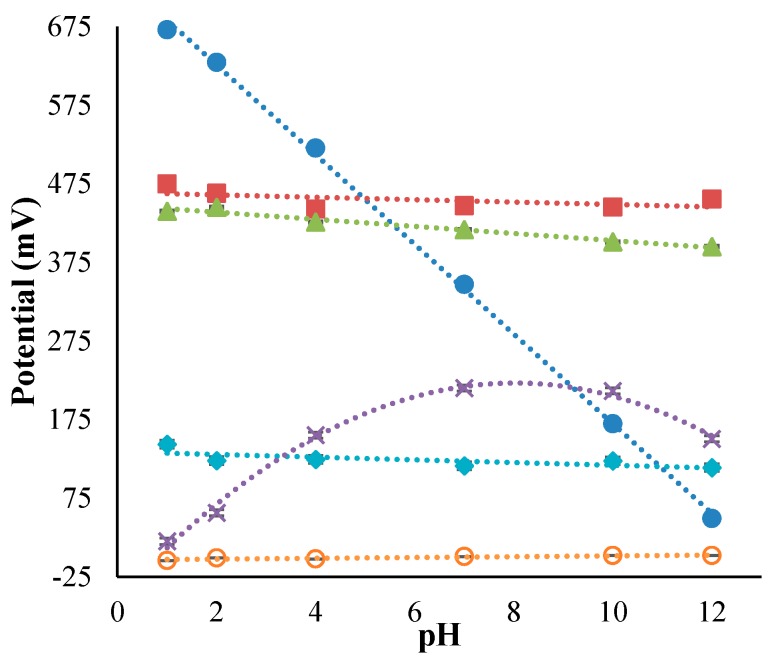

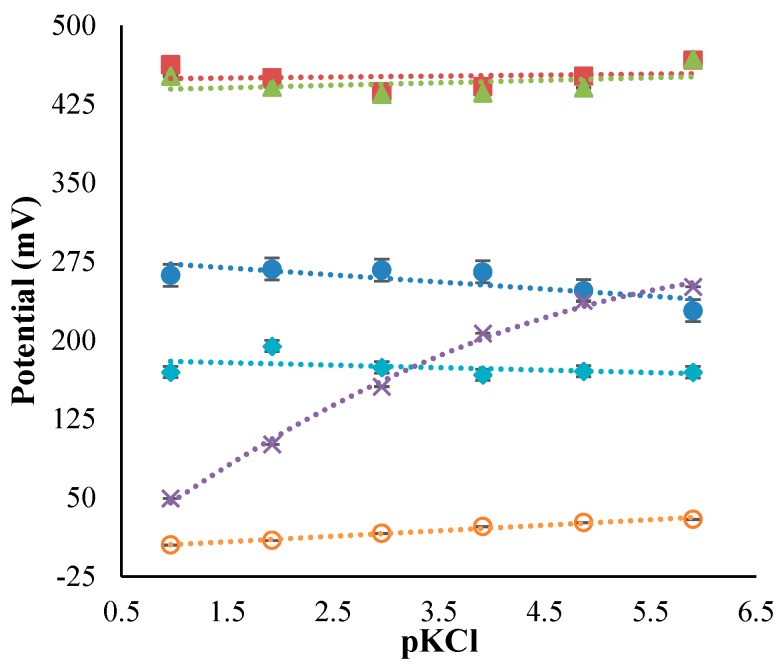
pH (**top**) and KCl (**bottom**) sensitivity of manufactured electrodes; 500 nm RuO_2_ (Blue Dots), RuO_2_-SiO_2_ (Red Squares), RuO_2_-SiO_2_-KCl (Green Triangles), Glued-RuO_2_ (Aqua Diamonds), Quasi-AgCl (Purple Crosses), and AgCl-KCl (Orange Circles).

All RuO_2_ electrodes were conditioned in pH 7 buffer for 24 h before use; the AgCl-KCl electrode was conditioned in 3 M KCl for 24 h before use; and the Quasi-Ag-AgCl electrode was conditioned in pH 7 buffer for 5 min before use (to prevent loss of AgCl). The pH and KCl sensitivity for each of the manufactured electrodes was examined by recording their responses against a commercial glass double junction Ag|AgCl|KCl reference electrode (Sigma). A summary of approximate pH and KCl sensitivity values for the manufactured electrodes is shown in Table 2. Figure 2 shows pH and KCl sensitivities of the manufactured electrodes. It is obvious from Table 2 and Figure 2 that all electrodes, apart from the Quasi-Ag|AgCl, exhibit low pH sensitivity between pH 1 and 12, compared to the RuO_2_ electrode, and that all electrodes apart from the Quasi-Ag|AgCl exhibit a relatively low response to KCl concentration. It should be noted that the non-linear pH response observed for the Quasi-Ag|AgCl electrode could be due to changes in chloride concentration between the commercial pH buffers used. In contrast, the sensitivity of the RuO_2_-junciton electrodes could be due to an inherent liquid junction potential, due to the junction material. These results show that, apart from Quasi-Ag|AgCl, all reference electrodes manufactured here could potentially be paired with a RuO_2_ working electrode for the development of an accurate pH sensor.

### 4.2. pH Sensor Performance

**Figure 3:**
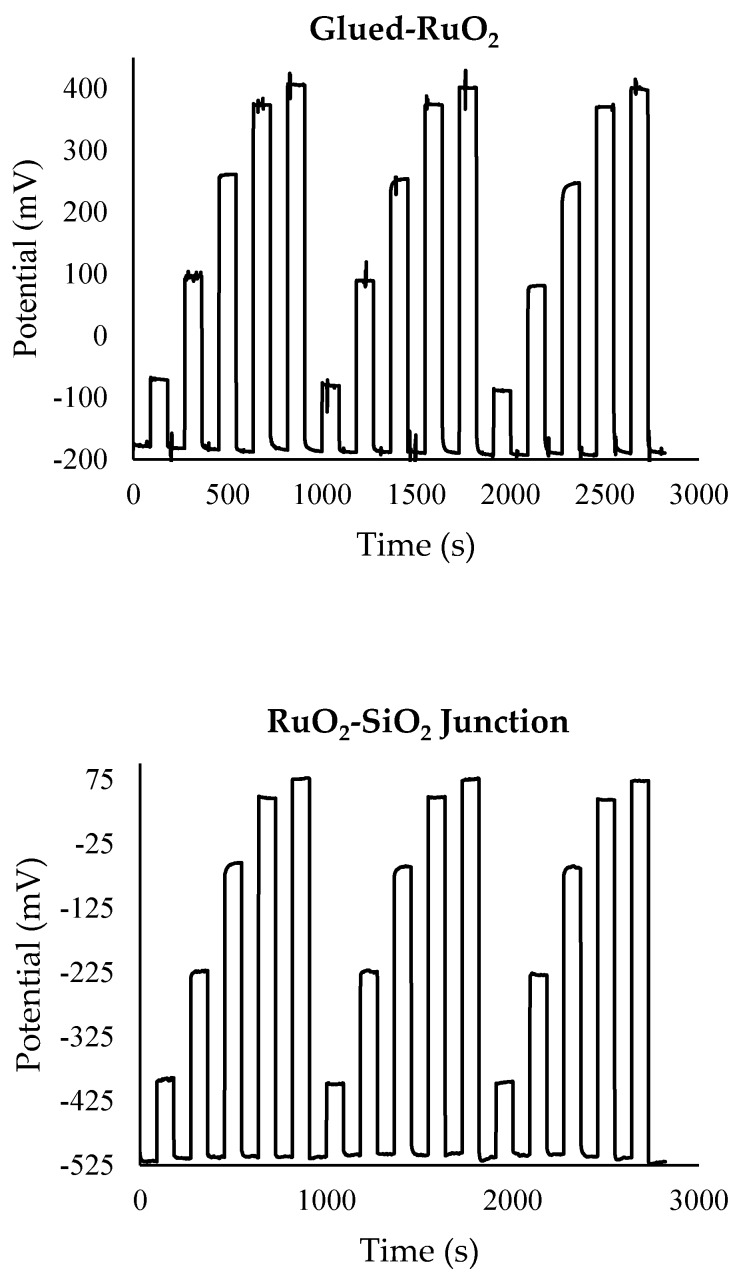

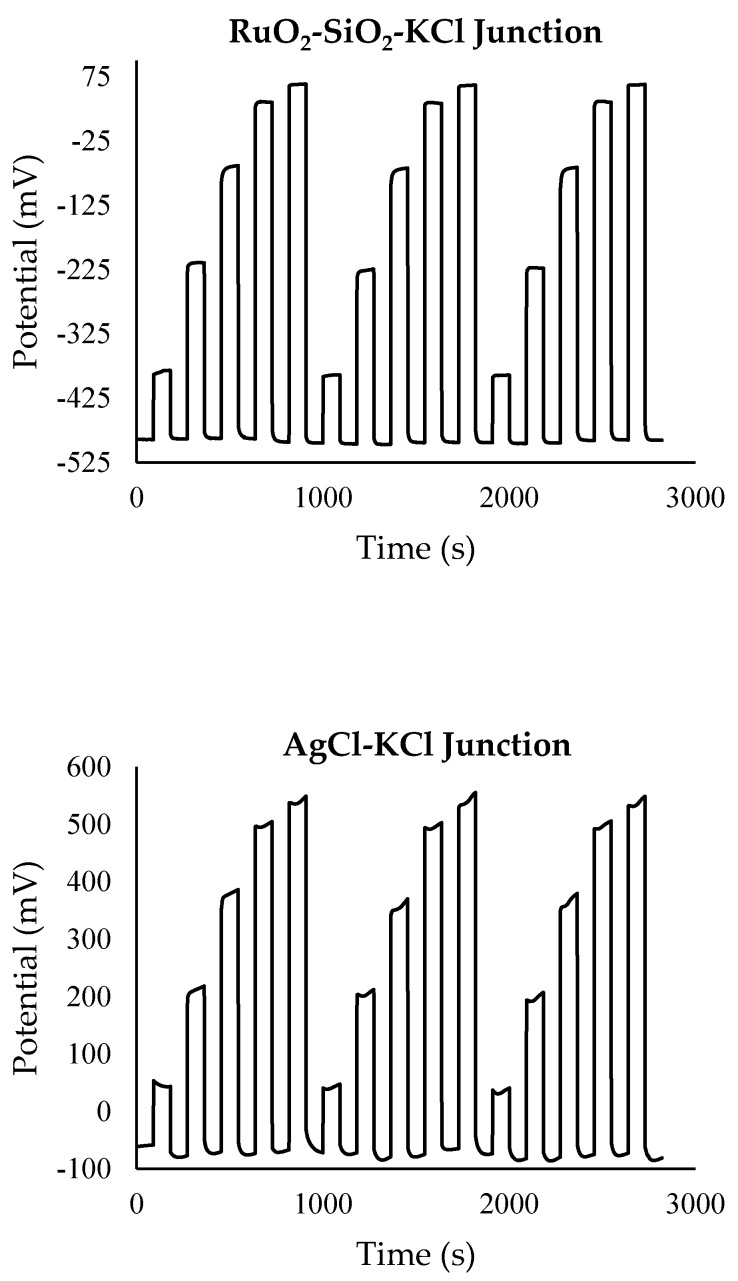
pH 12–10–7–4–2–1 data loops, with pH 12 between each measurement, for RuO_2_ pH sensors with Glued-RuO_2_, RuO_2_-SiO_2_, RuO_2_-SiO_2_-KCl, and AgCl-KCl reference electrodes.

Due to their low sensitivities to pH and KCl, the Glued-RuO_2_, RuO_2_-SiO_2_, RuO_2_-SiO_2_-KCl, and AgCl-KCl electrodes were paired with a RuO_2_ working electrode, giving four pH sensors. The effect of ageing, drift, and hysteresis were minimized by conditioning the sensors in a pH 7 buffer overnight, then equilibrating in a pH 12 buffer for 1 h before use [7]. The pH sensitivity, *E*^0^, linearity, hysteresis, and drift of these sensors was then examined by measuring sensor response when looped from pH 12–10–7–4–2–1.5 three times, with pH 12 between each step. Each pH data loop is presented individually in Figure 3, whilst the pH sensing properties are summarized in Table 3. 

The sensor employing an AgCl-KCl reference electrode exhibited the highest sensitivity (58.1 mV/pH), which is close to the theoretical maximum of 58.6 mV/pH, and agrees with previously reported results for this type of RuO_2_ working electrode against a glass double junction Ag|AgCl|KCl reference electrode [30]. However, the potential drifted during individual pH readings for this sensor, resulting in higher hysteresis and a larger drift over the experimental period. The sensors employing RuO_2_-SiO_2_ and RuO_2_-SiO_2_-KCl-based reference electrodes exhibited lower sensitivities to pH than the AgCl-KCl-based sensor. However, such sensors exhibited short reaction times (<30 s) (as shown in Figure 3) and a higher degree of stability, resulting in much lower hysteresis and drift values. The Glued-RuO_2_ pH sensor showed comparable results to the RuO_2_-SiO_2_ sensor; however, it was prone to electrical noise (Figure 3) and also exhibited a higher drift. 

**Figure 4:**
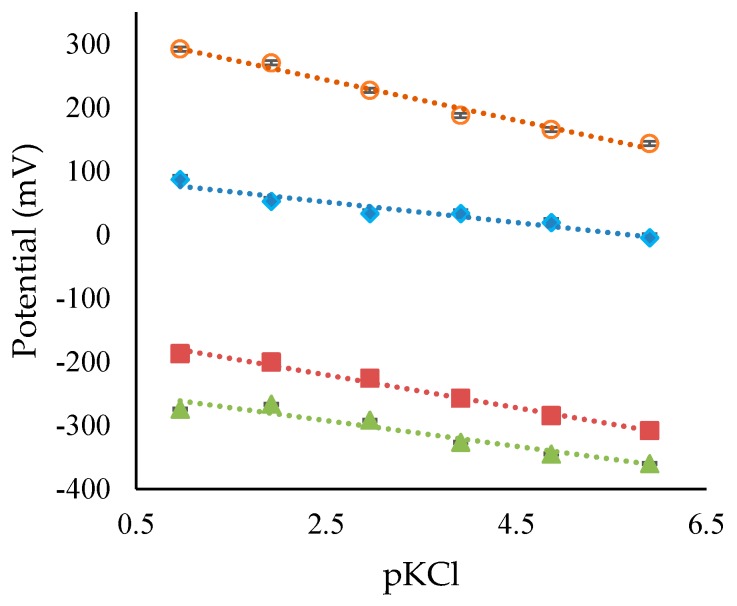
KCl sensitivity of RuO_2_ pH sensors with, RuO_2_-SiO_2_ (Red Squares), RuO_2_-SiO_2_-KCl (Green Triangles), Glued-RuO_2_ (Aqua Diamonds), and AgCl-KCl (Orange Circles) reference electrodes.

Table 4 and Figure 4 summarize the sensitivities to KCl observed for the sensors. The sensitivities to KCl were much higher than the expected 10 mV/pKCl, based on estimations using the data presented in Section 4.1. The change in potential observed as the KCl concentration increases can be attributed to both the working and reference electrodes. Firstly, RuO_2_ is known to respond to changes in ionic strength [5]. Secondly, a liquid junction potential could form at the reference electrodes due to the slow migration of compounds (impurities) in the various junctions. Lastly, the test solutions were un-buffered and some change in pH could occur during the addition of KCl. Based on this data, the RuO_2_-SiO_2_-based sensor displayed the best performance, in terms of acceptable sensitivity, low hysteresis, and low drift, compared to the other pH sensors.

### 4.3. Sample Solution Analysis

All developed pH sensors were evaluated in several sample matrices, including a 10 g/L solution of household borax (Borax), water sampled from a local lake (Lake), water sampled from the local beach (Sea), a common brand of cola (Cola), household vinegar (Vinegar), gastric dissolution media without enzyme (Gastric) (from Sigma), a pasteurized orange fruit drink (OJ), and a local lager beer (Beer). The samples were de-gassed where needed and allowed to equilibrate at room temperature before measurement. 

**Figure 5:**
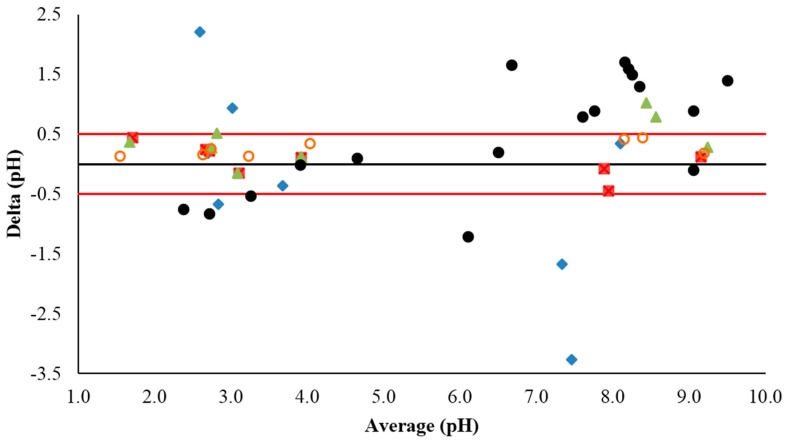
Bland-Altman plot for the pH sample data for RuO_2_ pH sensors with RuO_2_-SiO_2_ (Red Squares), RuO_2_-SiO_2_-KCl (Green Triangles), Glued-RuO_2_ (Aqua Diamonds), and AgCl-KCl (Orange Circles) reference electrodes. Compared with sample data from the work by J. Noh et al. [25] (Black Dots). Red lines denote ±0.5 pH units from the “true” pH value.

Table 5 shows the pH values measured by a commercial glass pH sensor (Eetech pH700, Thermo Scientific, Singapore), which were used as the “true” value for comparison with the data collected from the different pH sensors. The average difference (error) between the “true” and measured values was calculated for each sensor. As mentioned earlier, the RuO_2_-Glued sensor exhibited large shifts in potential and instability in many of the samples, resulting in poor performance. The RuO_2_-SiO_2_-KCl sensor returned acceptable results for most of the samples; however, the readings for the water and vinegar samples showed significant errors. The RuO_2_-SiO_2_ and AgCl-KCl sensor showed similar results with an average difference of 0.23 and 0.25 pH units form the glass senor, respectively. These results are presented graphically in Figure 5 as a Bland Altman plot, where the red line denotes an error of 0.5 pH units. Additionally, Figure 5 displays results obtained by the differential pH sensor developed by J. Noh et al. [25]; it is clear that the RuO_2_-SiO_2_ sensor developed here outperforms the differential pH sensor developed by J. Noh et al. [25]. 

It should be noted that the analysis of certain samples, such as white wine and fresh citrus juice, was not feasible. This was due to the presence of ascorbic acid and other redox active compounds in these samples, such as preservatives. These types of compounds caused large shifts in potential due to the oxidization/reduction of the working electrode [7]. The results shown in Table 5 and Figure 5 demonstrate that a differential pH sensor based on a RuO_2_ working electrode and a RuO_2_ reference electrode with a SiO_2_-loaded PVB junction can function as a reliable pH sensor in certain sample matrices. 

## 5. Conclusions

A pH sensor employing a RuO_2_ pH sensitive working electrode and a SiO_2_-PVB junction-modified RuO_2_ reference electrode has been developed and its performance evaluated. Experimental results have shown that the developed pH sensor exhibits good sensitivity (55.7 mV/pH) with low hysteresis (2.7 mV) and drift (2.2 mV/h). Experimental results have also shown that, for a selection of sample matrices, the pH values measured by the developed sensor are in excellent agreement with those measured by a commercial glass pH sensor. The attractive features of the developed pH sensing structure open the way towards the development of cost-effective, high-performance, and robust pH sensors for various applications. 

## Figures and Tables

**Table 1:** Composition of the different polyvinyl butyral (PVB) solutions.

Solution	Methanol (mL)	PVB (mg)	NaCl (mg)	KCl (mg)	SiO_2_ (mg)
PVB^NaCl^	2.0	234	150	-	-
PVB^Sio2^	2.0	234	-	-	150
PVB^Sio2+KCl^	2.0	234	-	150	150

**Table 2:** Summary of approximate pH and KCl sensitivity values for the manufactured electrodes. Electrode names are color coded to match their respective data series in Figure 2.

Electrode	pH Sensitivity		KCl Sensitivity	
	mV/pH	R^2^	mV/pKCl	R^2^
RuO_2_	−57	0.999	−6.7	0.629
RuO_2_-SiO_2_	−1.5	0.304	0.9	0.020
RuO_2_-SiO_2_-KCl	−4.5	0.967	2.4	0.118
Glued-RuO_2_	−1.7	0.523	−2.3	0.178
Quasi-Ag|AgCl	14	0.557	43	0.964
AgCl-KCl	0.5	0.823	5.2	0.984

**Table 3:** Summary of pH sensor performance calculated from pH loop data.

Reference Electrode	Sensitivity (mV/pH)		*E*^0^ (mV)		R^2^	Hysteresis (mV)		Drift (mV/h)
Glued-RuO_2_	−56.2	±0.5	483	±7.3	0.9988	2.1	±0.7	28
RuO_2_-SiO_2_	−55.7	±0.6	160	±1.4	0.9980	2.7	±1.0	2.2
RuO_2_-SiO_2_-KCl	−52.8	±0.2	143	±0.5	0.9980	1.4	±0.7	7.6
AgCl-KCl	−58.1	±1.6	620	±19	0.9996	6.7	±2.4	31

**Table 4:** Summary of KCl sensitivity values for RuO_2_ pH sensors with RuO_2_-SiO_2_, RuO_2_-SiO_2_-KCl, Glued-RuO_2_, and AgCl-KCl reference electrodes.

Reference Electrode	Sensitivity (mV/pKCl)		*E*^0^		R^2^
Glued-RuO_2_	−16.2	±4.9	92.4	±21	0.93
RuO_2_-SiO_2_	−25.8	±0.8	−156	±6.2	0.99
RuO_2_-SiO_2_-KCl	−20.2	±2.1	−242	±14	0.93
AgCl-KCl	−31.8	±0.3	323	±2.7	0.98

**Table 5:** Summary of pH measurements using the developed pH sensors and comparison to a commercial glass pH sensor.

Sensor	Borax	Lake	Sea	Cola	Vinegar	Gastric	OJ	Beer	Average Error
Glass pH Senor	9.1	8.2	7.9	2.6	2.6	1.5	3.2	3.9	±0.04
Glued-RuO_2_	5.8	6.5	8.3	2.8	3.5	3.7	2.5	3.5	±1.2
RuO_2_-SiO_2_	9.2	7.7	7.9	2.8	2.8	1.9	3.0	4.0	±0.23
RuO_2_-SiO_2_-KCl	9.4	9.0	9.0	2.9	3.1	1.9	3.0	4.0	±0.44
AgCl-KCl	9.3	8.6	8.4	2.9	2.7	1.6	3.3	4.2	±0.25
